# Wintersportverletzungen am Kniegelenk

**DOI:** 10.1007/s00132-022-04317-7

**Published:** 2022-10-14

**Authors:** Alexander Rauch

**Affiliations:** 1 ECOM – Praxis für Orthopädie, Sportmedizin & Unfallchirurgie, Arabellastraße 17, 81925 München, Deutschland; 2 Sporttraumatologie und Kniechirurgie, ATOS-Klinik München, Effnerstraße 38, 81925 München, Deutschland

**Keywords:** Vorderes Kreuzband, Skifahren, Snowboarden, Eishockey, Kreuzband-Ersatz/-Refixation, Anterior cruciate ligament, Skiing, Snowboarding, Ice-hockey, Acl recontruction/refixation

## Abstract

Wintersport stellt aufgrund der großen Beliebtheit eine relevante Entität für Knieverletzungen dar. Beim alpinen Skisport und beim Snowboarden ist das Kniegelenk die von Verletzungen hauptbetroffene Körperregion, beim Eishockey ist es die am dritthäufigsten betroffene Körperregion. Diverse Unfallmechanismen führen zu unterschiedlichen Verletzungsarten und -schweren. Neben Innenbandverletzungen sind Verletzungen des vorderen Kreuzbands von besonderer Bedeutung. Im Profisport sind teils schwere Kombinationsverletzungen gehäuft. Die Therapie wird am Beispiel der Ruptur des vorderen Kreuzbands exemplarisch dargelegt. Goldstandard ist die Ersatzbandplastik. Die „Return-to-sport“-Rate liegt mit 80 % für Skifahren und Snowboarden auf vergleichbarem Niveau mit Sommersportarten wie Football, Basketball oder Baseball. Für Eishockey ist sie mit 96 % noch besser. Prävention kann durch gezielte Trainingsprogramme aber auch durch Optimierung des Materials und dessen Einstellung erzielt werden.

## Abstract (en)

Winter sports represent a relevant entity for knee injuries due to their great popularity. In alpine skiing and snowboarding, knee joint injuries are the most common affected body regions, while in ice hockey they are in third place. Various accident mechanisms lead to different injury types and severities. In addition to medial collateral ligament injuries, anterior cruciate ligament injuries are of particular importance. In professional sports, severe combination injuries are more common. Therapy is exemplified using the anterior cruciate ligament rupture. The gold standard is replacement ligament surgery. The return-to-sport rate of 80% for skiing and snowboarding is comparable to summer sports such as football, basketball or baseball. For ice hockey, it is even better. Prevention is possible by targeted training programs, but also by optimizing the equipment and its adjustment.

Knieverletzungen gehören im Wintersport zu den häufigsten Verletzungsmustern. Dabei ist neben dem Innenband das vordere Kreuzband am stärksten betroffen. Allerdings ist die „Return-to-sport“-Rate hoch. In diesem Artikel werden die Epidemiologie, Diagnostik und Therapie ausführlich vorgestellt und es werden Hinweise zur Prävention gegeben.

## Einleitung

**Figure 1:**
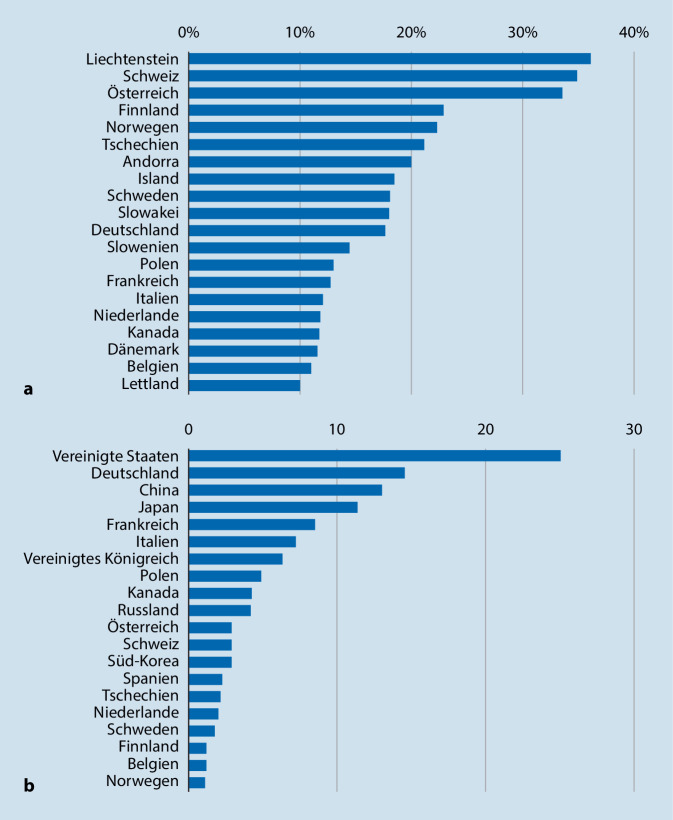

Verletzungen spielen generell im Sport eine große Rolle. Der Wintersport erfreut sich nicht nur in der Alpenregion, sondern auch im restlichen Deutschland großer Beliebtheit. Einen besonderen Stellenwert nimmt hier das alpine Skifahren ein. Jeder 6. Deutsche fährt mindestens gelegentlich Ski. Damit stellt Deutschland nach den USA und vor China die absolut gesehen zweitgrößte Skifahrerpopulation ([46]; Abb. 1).

Zu Sportverletzungen im Allgemeinen findet man fast 100.000 Einträge in der PubMed-Datenbank. Das Kniegelenk ist in vielen Sportarten eine, teilweise sogar die hauptbetroffene Körperregion. Spezifiziert man die Suchparameter wie in Tab. 1 angegeben, so lässt sich feststellen, dass wissenschaftliche Untersuchungen zu Verletzungen im Skisport schon seit langer Zeit durchgeführt werden und hier die Datenlage auch am größten ist. Es finden sich fast dreimal so viele Publikationen für das Suchwort „skiing“ (611 Treffer) als für dessen Überbegriff „winter sports“ (219 Treffer). Die auf das Kniegelenk nächstrangig bezogenen untersuchten Wintersportarten sind Eishockey (170 Treffer) und Snowboarden (67 Treffer).

**Table 1:** 

Suchbegriffe	Jahr der ersten Publikation	Anzahl der Ergebnisse ab 2012	Anzahl der Ergebnisse
Injury sports	1936	59.032	94.750
Injury winter sports	1954	105	219
Knee injury winter sports	1982	21	38
Knee injury skiing	1955	203	611
Knee injury snowboarding	1989	30	67
Knee injury (ice) hockey	1977	100	170

Haben früher schwere Knieverletzungen in den meisten Fällen im professionellen Sport das Karriereende bedeutet, so hat sich dies durch die stetig verbesserte medizinische Diagnostik und Therapie in den letzten Jahren geändert. Nichtsdestotrotz sind Kniegelenkverletzung beispielsweise beim Fußball der Hauptgrund für ein vorzeitiges Karriereende [22, 30]. Auch im Breitensport zeigt sich die Bedeutung z. B. anhand von jährlich über 40.000 durchgeführten Operationen des vorderen Kreuzbands (VKB) in Deutschland. Bei den Wintersportverletzungen nimmt die Verletzung des VKB eine (anatomisch wie auch im übertragenen Sinne) zentrale Rolle ein. Aufgrund der Häufigkeit und Relevanz dieser Verletzung wird in diesem Beitrag beispielhaft daran deren Therapie dargelegt, da aufgrund der Vielzahl der weiteren Verletzungsmöglichkeiten eine umfassende Abhandlung mit allen Therapiemodalitäten nicht möglich ist.

## Epidemiologie

Im Eishockey-Profisport liegen Knieverletzungen bezogen auf ihren Schweregrad (nach Ausfalltagen und direkten Verletzungskosten) an zweiter und bezogen auf die Häufigkeit an dritter Stelle. Rund drei Viertel der Verletzungen treten im Wettkampf auf und gut 25 % entstehen durch gegnerische Fouls. Die am häufigsten betroffene Region ist der Kopf, gefolgt vom Oberschenkel und Kniegelenk. Fast 80 % der Eishockey-Erstligaspieler verletzen sich mindestens einmal pro Saison [21].

Die relativ höchste Prävalenz für Kniegelenkverletzungen bei Wintersportprofis findet sich im Skisport in allen Disziplinen wieder: alpiner Skilauf (41,3 %), Freestyle (32,1 %), Skispringen (41,6 %) und auch beim Snowboarden (16,1 %).

Hier liegt das Verhältnis der Verletzungshäufigkeiten zwischen Wettkampf und Training etwa bei eins zu eins [18].

Im Breitensport liefert die jährliche ASU-Analyse der ARAG-Versicherung in Zusammenarbeit mit der Stiftung Sicherheit im Skisport eine große Datenbasis [38]. Hier zeigt sich seit Jahren das Kniegelenk als die am häufigsten verletzte Körperregion, zuletzt mit 33,1 % in der Saison 2019/2020. Auffallend ist hier ein ausgeprägter Unterschied zwischen Männern (21,6 %) und Frauen (50,5 %) [38, 40]. Das VKB ist bei mehr als der Hälfte aller operativ versorgten Knieverletzungen zumindest mitbeteiligt [10] und nimmt somit einen besonderen Stellenwert ein. Das Innenband (mediales Kollateralligament [MCL]) folgt in der Rangfolge der häufigsten Verletzungen beim Skifahren dem VKB nach [20].

Im Vergleich zum alpinen Skisport ist die Verletzungshäufigkeit am Kniegelenk beim Snowboarden um etwa die Hälfte geringer [8]. Beim Snowboarden führen MCL-Läsionen vor VKB-Läsionen die Verletzungen am Kniegelenk an.

**Figure 2:**
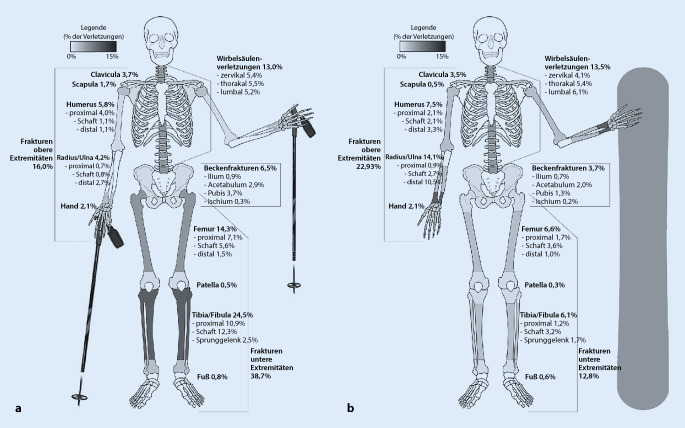

In einer großen Studie [5], die Wintersportverletzungen bei Skifahrern und Snowboardern untersucht hat, zeigte sich, dass es bei der Verletzungsschwere („injury severity score“ [ISS]) zwischen beiden Sportarten keine signifikanten Unterschiede gab. Korrelationen mit einem erhöhten ISS konnten für Altersgruppen von 18–29 sowie 60–69, männlichem Geschlecht und einem positiven Nachweis von Blutalkohol bzw. berauschenden Substanzen nachgewiesen werden. Frakturen machen mit 61 % über beide Gruppen die häufigste Verletzungsart aus; hierin war die häufigste Verletzungsregion (27 %) die untere Extremität (Abb. 2).

## Verletzungsmechanismen

### Eishockey

Beim Eishockey kann man zwei Pathomechanismen als wesentliche Ursache für Verletzungen unterscheiden:

- „high-speed low-mass injuries“ (Kollision des Körpers mit dem Puck, dem Schlittschuh oder dem Stock)
- „low-speed high-mass injuries“ (Bodycheck und Kollisionen mit der Bande oder der Eisfläche) [48].

In den meisten Fällen liegt ein direkter Körperkontakt (50 %) gefolgt von Kollisionen – Bande, Eis, Puck, Schläger – (40 %) vor. Bei nur 10 % passieren die Verletzungen ohne Kontakt [1].

Die großen Kräfte, die in diesem Sport wirken, erklären sich durch die hohe Skating-Geschwindigkeit von bis zu 45 km/h bei gleichzeitig harter Begrenzung des Spielfeldes durch eine umlaufende Bande. Auch die Schussgeschwindigkeit des Hartgummipucks von bis zu 170 km/h und der Stock als Spielgerät kann immens auf den Körper der Sportler einwirken [16].

### Skisport (alpin)

Anders als beim Eishockey kommt es beim Skifahren zu den meisten Verletzungen ohne Fremdkontakt, auch wenn in der letzten ASU-Statistik von 2019/2020 Kollisionsunfälle einen neuen Höchststand von 20 % erreichten [38].

**Figure 3:**
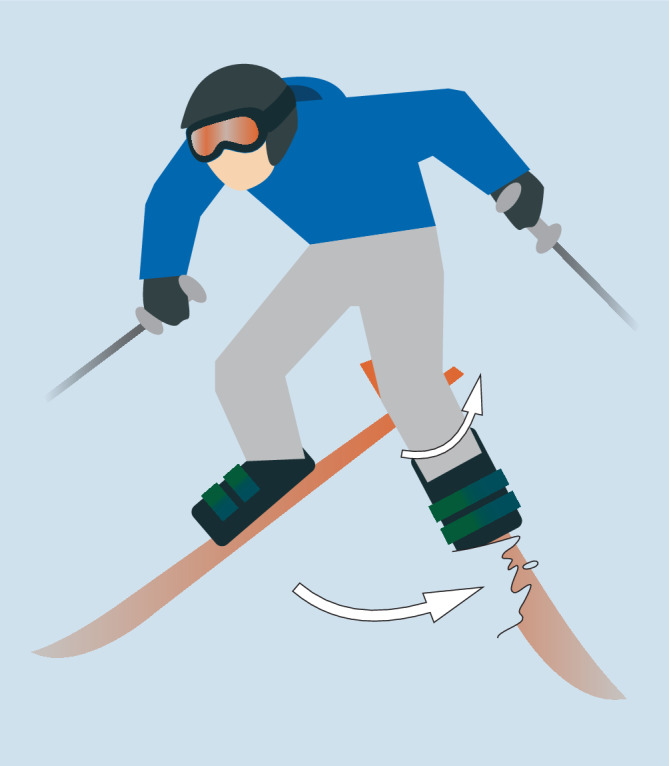

Der typische Unfallmechanismus für die Verletzung des VKB hat sich durch das geänderte Sportgerät (Carvingski) hin vom Rückwärtsdrehsturz zum Vorwärtsdrehsturz **(**Abb. 3**)** verschoben. Der Unterschied liegt darin, dass beim Rückwärtsdrehsturz externe Kräfte eine Innenrotation des Unterschenkels bei gebeugtem Kniegelenk verursachen, wohingegen es beim Vorwärtsdrehsturz durch externe Kräfte (Skier als Hebel) zu einer Außenrotation des Unterschenkels in Valgusstellung kommt [35].

**Figure 4:**
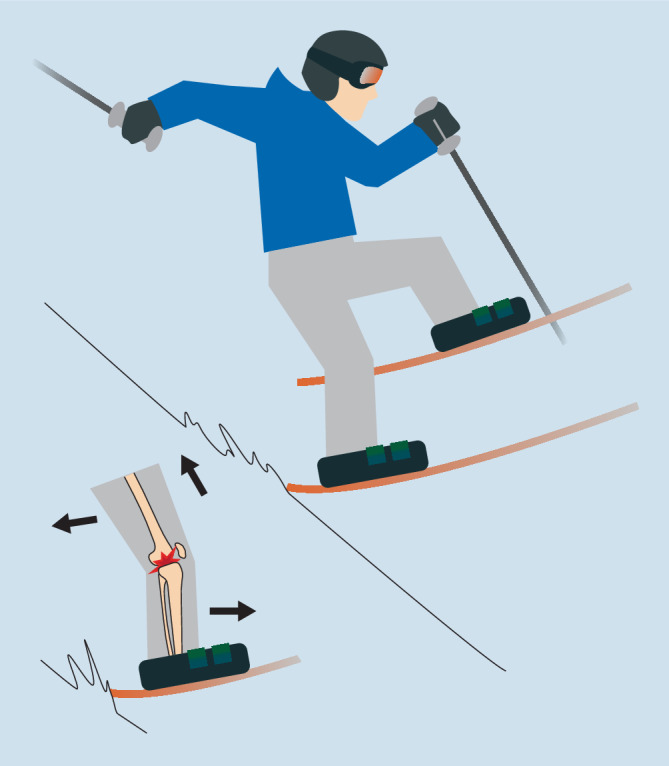

Ein weiterer typischer Mechanismus ist skischuhvermittelt **(**Abb. 4**)**. Hier führt eine vermehrte Rückenlage (z. B. beim Aufkommen nach einem Sprung oder einem falschen Beenden der Schwungphase mit zu weitem Abhocken nach hinten) zu einem Vorschub des Unterschenkels durch den Skistiefel bei durch die Gewichtskraft nach hinten gezogenem Oberschenkel [39].

Als weitere Variante, die vor allem bei schwerem Nassschnee vorkommt, führt ein plötzliches Innendrehen des Innenskis (z. B. an einem Schneehaufen) zu einem plötzlichen Stopp mit konsekutiver Überstreckung der Knie- und Hüftgelenke in resultierender Varusfehlstellung [45].

**Figure 5:**
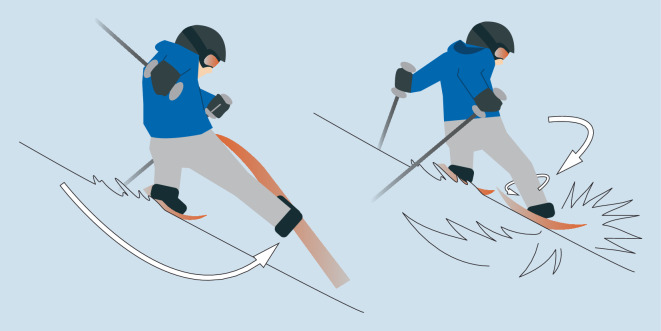

Beim Profisportler spielt der „Slip-catch“-Mechanismus **(**Abb. 5**)** eine Rolle. Hier kommt es nach einem Druckverlust des Außenskis zu einem Strecken des Kniegelenkes, um wieder den Kontakt zur Piste zu finden. Beim dann folgenden abrupten Greifen der Innenkante auf der Piste kommt es bei nun fast gestrecktem Kniegelenk zu einer Kompression zurück in die Flexion mit begleitender Innenrotation des Unterschenkels und Valgusfehlstellung [6, 7].

Kollateralbandverletzungen entstehen durch die üblichen Varus‑/Valgusstresstraumata, Frakturen meist durch direkten Anprall oder Landungen nach Sprüngen. Eine weitere Verletzungsquelle sind Zusammenstöße mit anderen Skifahrern oder Snowboardern. Hier sind schwerere Verletzungen zu beobachten als bei interindividuellen Kollisionen zwischen Snowboardern alleine [47].

### Snowboarden

**Figure 6:**
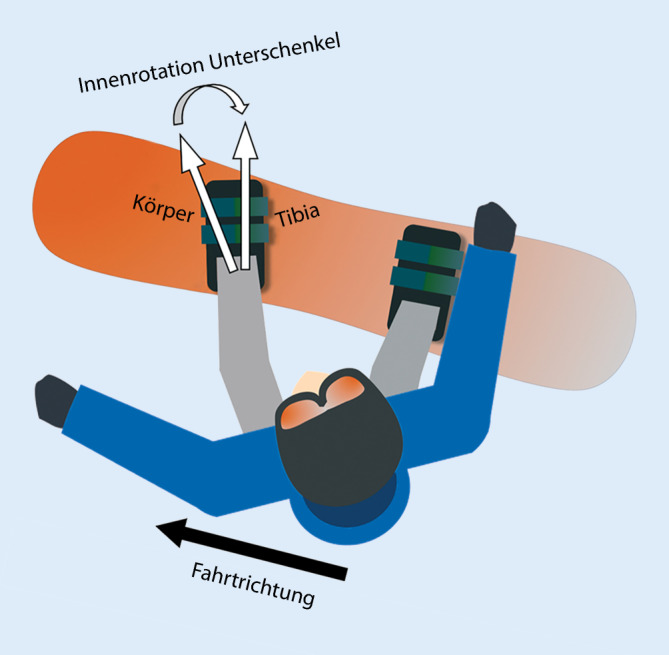

Da beim Snowboarden beide Beine mit nichtauslösbaren Bindungen am Board fixiert sind, werden Rotationskräfte auf das Kniegelenk signifikant reduziert und somit viele Kreuzbandverletzungen vermieden [7, 37]. Beim Snowboarden sind daher Kreuzbandverletzungen rund zehnmal seltener als beim alpinen Skisport [20]. Im Profisport ist dieses Verhältnis aufgrund der Höhe und dem Impact bei den immer spektakulärer durchgeführten Sprüngen merklich reduziert. Das Springen selbst ist auch im Breitensport eine der Hauptursachen für Kreuzbandverletzungen. Ursächlich hierfür ist das Landen auf flachem Terrain mit leicht flektiertem Kniegelenk und gleichzeitig exzentrischer Anspannung des M. quadriceps femoris. Die dadurch entstehende Tibiatranslation nach anterior kann mit begleitender leichter Valgusfehlstellung und Innenrotation des Unterschenkels (durch axiale Kompressionskräfte bei der Landung) ausreichen, eine VKB-Ruptur herbeizuführen [9, 11]. Auch die Stellung des führenden Beins auf dem Snowboard begünstigt eine Verletzung des VKB. Obwohl der führende Fuß in leichter Außenrotation auf dem Brett fixiert ist, kommt es durch die Körperdrehung in Fahrtrichtung über diese Außenrotation hinaus zu einer resultierenden Innenrotation des Unterschenkels **(**Abb. 6**)**. Bei gleichzeitig voller Streckung im Kniegelenk ist dies eine nachgewiesene Risikolast auf das VKB [11]. Das führende Knie ist grundsätzlich anfälliger für Verletzungen.

Auch Kollisionen zwischen Snowboardern können Bandverletzungen am Kniegelenk nach sich ziehen. Am häufigsten kommt es in Snowparks zu diesen Ereignissen, wobei meist einer der beiden Kollisionspartner steht oder sitzt/kniet [47].

Im Breitensport können vor allem bei Anfängern Bandverletzungen beim Liftfahren auftreten, da hier die noch ungeübten Sportler nur mit einem Bein am Snowboard fixiert sind. Verkantet sich das Board während der Fahrt im Schlepplift oder beim Aussteigen aus dem Sessellift, können hier Torsionskräfte und Valgusfehlstellungen auftreten, die VKB- oder MCL-Rupturen bedingen können.

## Verletzungsmuster

Die Läsion des medialen Kollateralbandes (Innenband) ist die häufigste Verletzung des Kniegelenkes beim Eishockey [33]. Verletzungen der Kreuzbänder sind deutlich seltener und treten im Vergleich zu anderen Sportarten wegen der oft massiv einwirkenden Kräfte gehäuft als (Sub‑)Luxationstraumata auf [48].

Das Verletzungsmuster ist im Skisport aufgrund der Vielzahl an möglichen Unfallmechanismen durch Stürze, Kollisionen oder Crash-Landungen nach Sprüngen breit gefächert.

Bei den 447 Knieverletzungen, die im Skiweltcup auftraten, wurde in 168 Fällen eine Ruptur des VKB festgestellt [18]. In einer Fallserie von 28 Profirennläufern, die sich einer VKB-Rekonstruktion unterzogen haben, lagen Begleitverletzungen in Form von 29 % MCL-Läsionen, 54 % Knorpelläsionen und 82 % Meniskusläsionen vor. In 22 % der Fälle lag sogar eine beidseitige VKB-Läsion vor [19]. Im Breitensport sind diese Kombinationsverletzungen insgesamt deutlich seltener.

Beim Snowboarden ziehen sich Anfänger gehäuft Innenbandverletzungen am Kniegelenk zu. Dahingegen verletzt sich der Profisnowboarder (Freestyle) vor allem das VKB [9].

In bis zu einem Drittel aller Verletzungen bei Skifahrern und Snowboardern kommen tiefe Schnittwunden als isolierte Verletzungen oder Begleitverletzungen vor. Der überwältigende Großteil von 94 % dieser Verletzungen tritt zwischen Knie und Hüfte auf und wird durch die scharfen Kanten oder Spitzen der Ski bzw. des Snowboards verursacht. Skifahrer sind mit über 90 % am häufigsten betroffen. Die Ursachen liegen zumeist bei einem ungewollten Auslösen der Skibindung, unzureichend technischem Fahrkönnen und Zusammenstößen [41]. In der Regel konnte auch eine adäquate Kleidung diese Verletzungen nicht verhindern.

## Diagnostik und Therapie am Beispiel der VKB-Ruptur

Zunächst sollte im Rahmen der Anamnese der genaue Verletzungsmechanismus erfragt werden. Hier können schon erste Hinweise auf mögliche Verletzungsarten erfolgen. Die klinische Untersuchung sollte gezielt und umfassen nach den üblichen Standards erfolgen [24].

### Konventionelles Röntgen

In der Akutversorgung von Knieverletzungen sollten zunächst konventionelle Röntgenaufnahmen des Kniegelenkes in 2 Ebenen (a.-p. und seitlich) durchgeführt werden. Hier können sich schon indirekte Zeichen einer VKB-Ruptur wie z. B. die Segond-Fraktur zeigen. Diese ist eine Avulsionsfraktur des anterolateralen Ligamentes an der anterolateralen Tibia, meist vergesellschaftet mit einer VKB-Ruptur. Zudem können knöcherne VKB-Ausrisse, häufiger bei Kindern, erkannt werden.

### Kernspintomographie (MRT)

**Figure 7:**
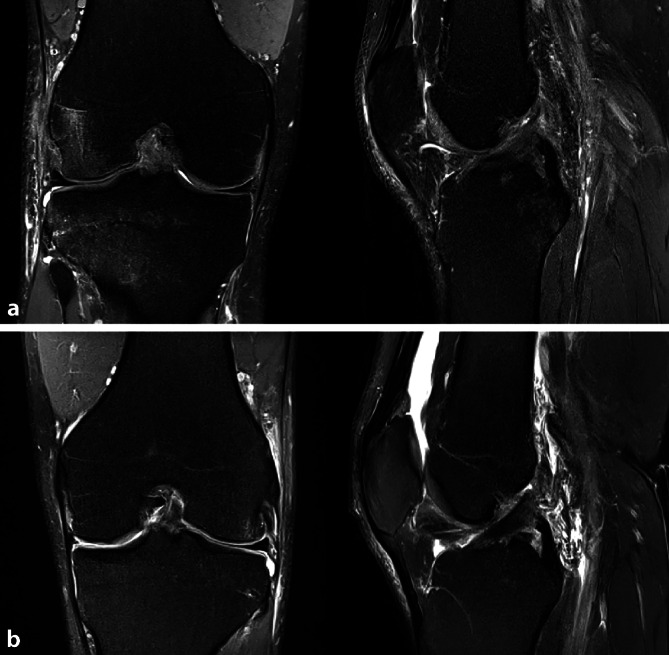

Die MRT-Untersuchung ist zur Abklärung einer VKB-Verletzung die Methode der Wahl **(**Abb. 7**)**. Direkte Zeichen einer VKB-Ruptur sind im Wesentlichen Kontinuitätsunterbrechungen, fehlender Nachweis des Bandes in anatomischer Position, wellige Kontur des VKB, Verlagerung des tibialen oder femoralen Bandansatzes bzw. Auftreibung oder diffuse Signalstörung des VKB. Des Weiteren können in der MRT Begleitverletzungen wie Knorpelverletzungen, Meniskusrisse und weitere Bandverletzungen abgeklärt werden.

### Computertomographie (CT)

Eine native CT-Abklärung kann bei begleitenden Frakturen sinnvoll sein.

### Begleitverletzungen

#### Meniskus(rampen)läsionen

Da Meniskusverletzungen Prädiktoren für eine folgende Gonarthrose sind [34] und deren Vorliegen insbesondere die Rotationsläxizität verstärkt [29, 30], sollte immer eine begleitende Therapie der Meniskusverletzungen erfolgen. Auf die Meniskusrampe sollte insbesondere intraoperativ ebenso geachtet werden, da diese sich derzeit mittels MRT nicht suffizient darstellen lässt. Gemäß aktuellen Untersuchungen treten jedoch Meniskusrampenläsionen in bis zu 20–30 % bei VKB-Rupturen auf [28, 43].

#### Mediale Kollateralbandläsion

Bei Läsionen des medialen Kapselbandkomplexes kommt es unter Außenrotation der Tibia zu einer vorderen Subluxation des medialen Plateaus. Anhand von biomechanischen Studien konnte die Relevanz der einzelnen Innenbandstrukturen (tiefes Innenband, oberflächliches Längsband und hinteres Schrägband) untersucht und daran die anteromediale Instabilität klassifiziert werden [43]. Spätestens bei Grad-III-Läsionen sollte eine mediale Rekonstruktion begleitend zur VKB-Rekonstruktion erfolgen [24].

#### Laterale Kollateralbandläsion

Laterale Kollateralbandverletzungen treten meist in Kombination mit Verletzung der zentralen Bandstrukturen auf. Partielle Rissbildungen ohne höhergradige Instabilität können konservativ behandelt werden. Jedoch sollte bei Komplettrupturen oder höhergradigen Instabilitäten eine Rekonstruktion begleitend zur VKB-Rekonstruktion erfolgen. Bei einer chronischen Instabilität muss diese ebenfalls mit adressiert werden. Hier stehen verschiedenen operative Verfahren wie z. B. die Stabilisierungsoperationen in der Larson-Technik, La-Prade-Technik oder Umlenkungs‑/Versetzungsoperationen wie der Popliteusbypass oder die Bizepstenodese nach Clancy zur Verfügung [24].

#### Unhappy Triad/Tetrad

Die Kombinationsverletzung von VKB, medialem Kollateralband und Innenmeniskus wird als Unhappy Triad bezeichnet [32]. Neuere Studien sprechen mittlerweile von einer Tetrade anstelle einer Triade, da in den meisten Fällen auch eine Läsion des anterolateralen Komplexes nachgewiesen werden kann [15].

#### Frakturen

Auch kniegelenknahe Frakturen können als Begleitverletzungen auftreten. Da diese eine eigene Entität aufweisen, wird an dieser Stelle auf folgenden Beitrag „Tibiakopf- und Tibiaschaftfrakturen im Wintersport“ von L. Kohn und A. Rauch verwiesen [23].

## Therapiealgorithmus am Beispiel des VKB

Rupturen des VKB können konservativ oder operativ behandelt werden. Eindeutige, klare, evidenzbasierte Empfehlungen zur Versorgung einer VKB-Ruptur liegen zum aktuellen Zeitpunkt nicht vor. Für den Therapieentscheid existieren jedoch Kriterien, die für oder gegen eine operative Therapie sprechen. Hier spielen das Patientenalter, das Aktivitätslevel, die Kniegelenksstabilität, die Begleitverletzungen und das Operationsrisiko eine Rolle. Der Zeitpunkt der Versorgung richtet sich auch nach den Begleitverletzungen.

Eine isolierte VKB-Ruptur kann entweder innerhalb der ersten 48 h nach dem Unfall oder nach Abklang der akuten traumatischen Entzündungsphase, meist nach ca. 4–6 Wochen, operativ versorgt werden. Das Kniegelenk sollte zum Zeitpunkt der Ersatzbandplastik abgeschwollen, reizlos und nicht mehr schmerzhaft sein. Zudem sollte eine Beugung von über 90 ° und eine nahezu komplette Streckung erreicht werden.

Ist ein VKB-Erhalt geplant, so wird eine Operation innerhalb von 2–3 Wochen nach dem Trauma empfohlen.

Relevante Begleitverletzungen benötigen in der Regel ebenfalls ein zeitnahes Vorgehen innerhalb von 2–3 Wochen. In diesen Fällen muss individuell abgewogen werden, ob eine simultane VKB-Rekonstruktion durchgeführt wird. Nach wie vor wird über eine erhöhte Arthrofibroserate nach einer VKB-Ersatzbandplastik während der posttraumatischen Entzündungsphase diskutiert. Jedoch konnte auch gezeigt werden, dass eine einzeitige Meniskusnaht in Kombination mit einer VKB-Rekonstruktion bessere Ergebnisse liefert als ein zweizeitiges Vorgehen.

**Figure 8:**
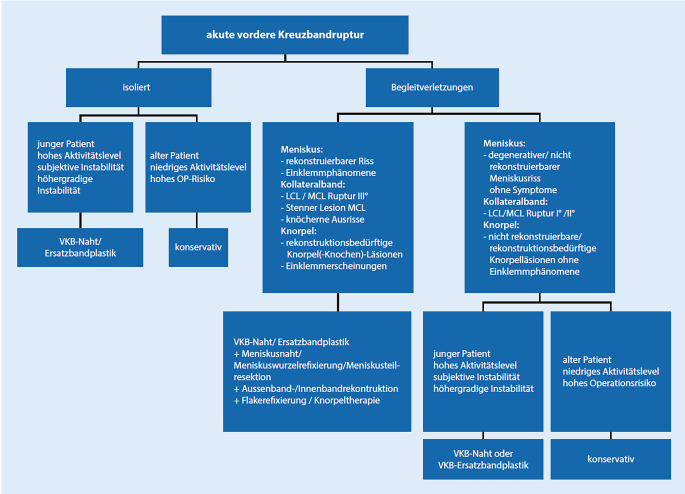

Ein Überblick zum Vorgehen bei akuter VKB-Ruptur ist in Abb. 8 dargestellt.

### Operative VKB-Rekonstruktion

Der Goldstandard der Kreuzbandrekonstruktion ist die Ersatzplastik mittels autologer Sehne. Am häufigsten werden im Primärfall die Hamstring-Sehnen verwendet. Jedoch erlebt die Quadrizepssehne in den letzten Jahren eine Renaissance und findet insbesondere im Revisionsfall aber auch bei zusätzlich medialer Knieinstabilität immer mehr Beliebtheit.

Durch Einlegen des Transplantates in einer Vancomycin-Lösung vor der Implantation wird das Infektionsrisiko signifikant verringert [4].

In einer Arbeit von Krutsch et al. konnte gezeigt werden, dass bei Skisportlern und Snowboarden (Wintersport) im Vergleich zu Fußballern (Sommersport) ein signifikant geringeres postoperatives Infektionsrisiko besteht [26].

### Operationstechniken

Bei der verbreitetsten Operationstechnik wird über ein anteromediales Arbeitsportal der femorale Bohrkanal angelegt, wodurch eine gute anatomische Positionierung des femoralen Bohrkanals ermöglicht wird. Auch wenn die anatomische Positionierung des femoralen Bohrkanals in dieser Technik nicht trivial ist, besteht der Vorteil, dass man eine freie, unabhängig voneinander positionierbare Anlage des tibialen und femoralen Bohrkanals durchführen kann. Hierdurch kann ein anatomischer Verlauf der Ersatzbandplastik erreicht werden.

**Figure 9:**
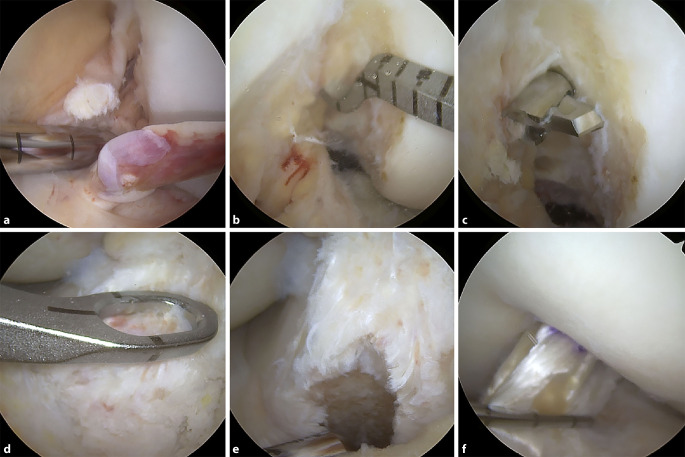

Varianten zu diesem Grundprinzip bestehen z. B. in der „Outside-in“- oder „All-inside“-Technik. In dieser Technik erfolgt die Anlage des femoralen Zielbohrdrahtes analog zum tibialen Bohrkanal von extraartikulär nach intraartikulär. Der Vorteil dieser Technik ist die freie Positionierung des femoralen Bohrkanals in 90 ° Flexion. Da hier keine Flexion über 110°, wie bei der anteromedialen Technik, notwendig ist, ist die Visualisierung der lateralen Notchwand meist besser. Durch die freie Positionierung besteht zudem die Möglichkeit einer anatomisch exakten femoralen Bohrkanalanlage. Durch spezielle retrograde Bohrer besteht die Möglichkeit einer knochensparenderen Bohrkanalanlage im Sinne von Sacklöchern femoral wie tibial. Vorteil dieser „All-inside“-Technik **(**Abb. 9**)** ist, dass sie meist schon ab einer Transplantatlänge von nur 60 mm durchführbar ist, wodurch in der Regel bei der Wahl von Hamstring-Transplantaten die Semitendinosussehne ausreicht und die Grazilissehne belassen werden kann.

Es besteht der Trend, möglichst viel des rupturierten Stumpfes zu erhalten oder sogar das vordere Kreuzband zu refixieren

Aufgrund neuerer histologischer Studien zum VKB besteht der Trend, möglichst viel des rupturierten Stumpfes zu erhalten. Hierdurch erhofft man sich eine verbesserte postoperative Propriozeption und durch den Erhalt des Synovialschlauches eine schnellere Vaskularisierung. Eine erhöhte postoperative Zyklopsrate durch das Belassen von Stumpfanteilen, wie es früher befürchtet wurde, konnte bis dato nicht nachgewiesen werden. Eine Überlegenheit der stumpferhaltenden VKB-Rekonstruktion im Vergleich zur stumpfresezierenden Technik konnte zwar aktuell noch nicht eindeutig gezeigt werden, jedoch sehen wir den Erhalt von Mechano- und Dehnungsrezeptoren sowie von Synovialgewebe mit mesenchymalen Stammzellen durchaus sinnvoll. Zudem können durch das Belassen der femoralen und tibialen Stümpfe die patientenindividuellen anatomischen Begebenheiten besser beachtet werden.

Durch modernere arthroskopische Techniken und Instrumente, aber auch durch ein verbessertes Verständnis, welche Kreuzbandrupturen erhaltungsfähig sind, erlebt der VKB-Erhalt aktuell eine Renaissance. Es scheinen sich frische femorale oder tibiale Avulsionsverletzungen mit guter Stumpfqualität gut für den Erhalt zu eignen, wohingegen intraligamentäre Bandrupturen oder eine schlechte Stumpfqualität gegen einen Erhalt sprechen. Zudem sollte die Versorgung zeitnah innerhalb von 2–3 Wochen nach dem Unfall erfolgen.

**Figure 10:**
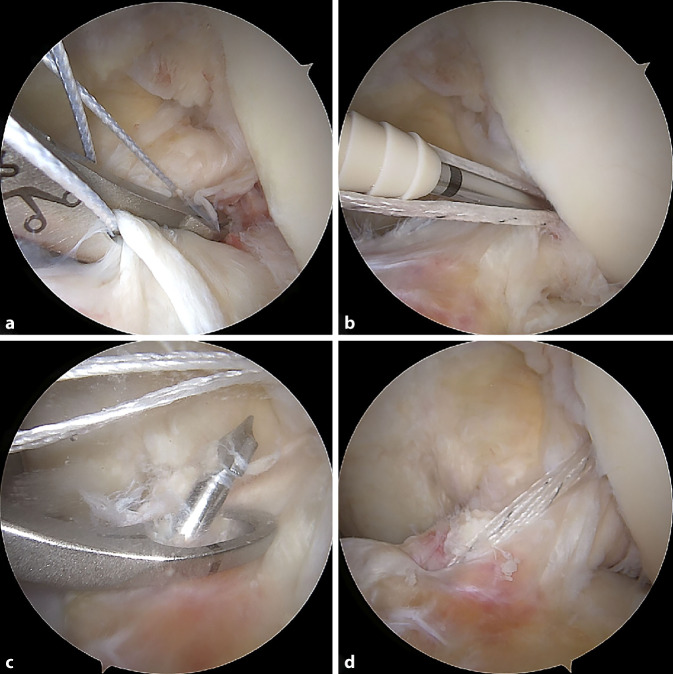

Über ein sogenanntes Ligament-Bracing kann zusätzlich eine statische Unterstützung des genähten oder refixierten VKB erzielt werden. Hierbei wird ein stabiles Fadenbandmaterial parallel zum VKB als interne Schienung („internal brace“) eingezogen. Durch das „internal bracing“ soll ein passager mechanischer Schutz der VKB-Naht bis zu deren sicheren Ausheilung gewährleistet werden **(**Abb. 10**)**.

Insbesondere in Fällen von komplexerer Instabilität scheint neben der Rekonstruktion des VKB eine zusätzliche anterolaterale Stabilisierung sinnvoll zu sein [17, 42, 44].

Wann eine zusätzliche anterolaterale Stabilisierung durchgeführt werden sollte, ist noch nicht endgültig geklärt. Die Empfehlung besteht jedoch aktuell bei höhergradigem positivem Pivot-Shift-Test, bei einer zusätzlich vorliegenden Segond-Fraktur, bei Re-Rupturen und bei Profi- oder Wettkampfsportlern mit pivotierenden Sportarten. Zudem kann eine anterolaterale Stabilisierung bei persistierender Rotationsinstabilität nach VKB-Ersatzplastik durchgeführt werden.

Bezüglich der Operationstechnik wird aktuell zwischen der nichtanatomischen extraartikulären Stabilisierung in der modifizierten Lemaire-Technik mittels Traktusstreifen und der anatomischen Rekonstruktion des anterolateralen Ligamentes mittels autologer Grazilissehne unterschieden.

### Konservative Therapie und Nachbehandlung

In aktuellen Analysen konnte die operative Kreuzbandersatzbandplastik einen Vorteil gegenüber der konservativen Therapie zeigen und wird insbesondere bei jungen sportlich aktiven Patienten aktuell empfohlen. Jedoch können verschiedene Faktoren wie z. B. Patientenalter oder -konstitution sowie mögliche Grund- oder Begleiterkrankungen eine konservative Therapie bedingen. Die konservative Therapie ähnelt der Nachbehandlung einer operativen Therapie. Aktuell wird immer mehr von den rein zeitbasierten Nachbehandlungsschemata Abstand genommen und zu einer kriterienbasierten Rehabilitation mithilfe von verschiedenen Funktionstests bzw. Testbatterien gewechselt. Um die jeweils nächste Behandlungsphase zu erreichen, sollten objektive Parameter durch Funktionstests herangezogen werden. Mit diesen „Return to activity/‑sport/‑play/‑competition“-Tests lässt sich so zum einen die Nachbehandlung individuell auf den Patienten und dessen jeweiligen Leistungsstand ausrichten und zum anderen das Risiko einer erneuten Verletzung verringern.

### „Return to sport“

Die Datenlage zu „Return-to-sport“ in Wintersportarten ist deutlich dünner als für Sommersportarten wie z. B. Fußball. Eine Studie von Erickson et al. [13] konnte in einer Fallserie für Snowboarder und Skifahrer für Profisportler die „Return-to-competition“(RTC)-Rate nach VKB-Rekonstruktion zu den X‑Games untersuchen. Insgesamt konnten 80 % wieder bei den X‑Games antreten, wobei die Rate bei den Skifahrern mit 87 % (13 von 15) im Vergleich zu Snowboardern mit 70 % (7 von 10) besser war. Die Medaillenausbeute lag bei den Skifahrern bei 22 (9 Gold, 5 Silber, 8 Bronze) vor dem Zeitpunkt der VKB-Rekonstruktion und bei 24 (16 Gold, 2 Silber, 6 Bronze) danach. Bei den Snowboardern konnte eine deutliche Verbesserung für die Zeit nach der Operation mit 19 Medaillen (7 Gold, 7 Silber, 5 Bronze) im Vergleich zu vor der Operation mit 7 Medaillen (4 Gold, 1 Silber, 2 Bronze) erreicht werden. Ein RTC auf Vorverletzungsniveau ist damit grundsätzlich möglich, wobei einer von 5 Athleten scheitert.

Für Eishockey ist die RTC-Rate auf National-Hockey-League-Niveau mit 96 % sogar noch besser [14].

## Prävention

Verletzungen des VKB haben weitreichende Folgen für einen sportlich aktiven Menschen. Eine chronische VKB-Instabilität führt zu einer signifikanten Zunahme von relevanten Meniskus- und Knorpelschäden. Eine VKB-Ersatzbandplastik reduziert diese Folgeschäden und damit die Arthrosprogredienz des Kniegelenkes. Die Rate der Patienten, die auf ihr altes sportliches Niveau kommen, beträgt jedoch nur ca. 65 % bei Hobbysportlern und 83 % bei Eliteathleten [3, 27, 31]. Diese Daten verdeutlichen die Relevanz, präventiv tätig zu werden. So wurden im Laufe der letzten Jahre verschiedene Präventionsprogramme entwickelt (z. B. Sportsmetrics, Prevent Injury and Enhance Performance [PEP], Knee Ligament Injury Prevention Program, FIFA 11+). Durch diese speziellen Programme können die Raten an VKB-Rupturen beim Fußball um 51 % reduziert werden [12]. Auch für den Skisport gibt es entsprechende Bestrebungen. Bei schwedischen „High-school“-Studenten konnten mit entsprechenden Präventionsprogrammen die Prävalenz von Kreuzbandrupturen von 8,1 % auf 3,9 % gesenkt werden [49]. Die im Profisport konsequente Datensammlung (Smartabase der Ski-Nationalmannschaft) wird eine Möglichkeit bieten, die gewonnenen Erkenntnisse auch auf den Breitensport zur Prävention zu übertragen (siehe den Beitrag „Wintersportnation Deutschland – Verletzungen im Ski-alpin-Rennsport und -Breitensport“ von M. Köhne und K. Waibel [25]).

Auch die richtige Auswahl des Materials/Sportgerätes kann einen positiven Einfluss auf die Reduktion der Verletzungswahrscheinlichkeit haben [36].

## Fazit für die Praxis

- Wintersport hat einen bedeutenden medizinischen Aspekt, da sich insbesondere Skifahren und Snowboarden großer Beliebtheit erfreut.
- Es gibt sowohl sportartspezifische Verletzungsmuster als auch Unterschiede zwischen Profi- und Breitensportlern.
- Verletzungen des vorderen Kreuzbands gehören zu den häufigsten Sportverletzungen am Kniegelenk im Wintersport.
- Die Therapie sollte in Abhängigkeit von Begleitverletzungen erfolgen.
- Zur Prävention gibt es neben speziellen Trainingsprogrammen auch den Bereich der Materialoptimierung.

## Abkürzungen

ASU: Auswertungsstelle für Ski-Unfälle
ISS: „Injury severity score“
LCL: Laterales Seitenband
MCL: Mediales Kollateralligament
RTC: „Return-to-competition“
VKB: Vorderes Kreuzband
